# Empirically assessing corporate adaptation and resilience disclosure using AI

**DOI:** 10.1038/s44168-025-00321-7

**Published:** 2026-02-12

**Authors:** Roberto Spacey Martín, Nicola Ranger, Tobias Schimanski, Markus Leippold

**Affiliations:** 1 London School of Economics and Political Science, London, UK; 2 Environmental Change Institute, University of Oxford, Oxford, UK; 3 University of Zurich, Zurich, Switzerland

**Keywords:** Climate sciences, Environmental sciences, Environmental social sciences, Environmental studies

## Abstract

The extent to which firms are adapting and building resilience to environmental change is crucial information for financial institutions, regulators and governments. While corporates’ physical climate risk exposure of their assets to environmental change can be calculated using models, additional information is needed to evaluate their vulnerability to physical climate change, how well they are adapting and broader alignment with societal adaptation and resilience (A&R) goals. This paper empirically evaluates the extent of A&R-related information in current corporate sustainability reports to provide such insights. We build on established sustainability disclosure frameworks and develop an A&R disclosure framework that we combine with the latest advances in large language models to assess S&P 500 company sustainability reports. We prove that corporate A&R information in sustainability reports is lacking, particularly around risks, metrics and targets, underlining the need to consider other data sources when assessing firm-level risks and contributions to societal A&R goals.

## Introduction

Irrespective of future emissions trends, there is a need to build resilience to the impacts of climate change and nature loss already locked in. Forty percent of the global population resides in highly climate-vulnerable areas^1^, and some systems are already reaching adaptation limits^2^, restricting the solutions space for effective responses^3^. Ranger and Oliver^4^ further demonstrate the significant dependencies on nature and the financial materiality of nature-related risks and their interlinkages to climate-related physical risks. Yet, adapting to climate change and building resilience to wider environmental risks has received considerably less attention than mitigation in the business and finance sphere, particularly when considering the status of the policy architecture and the amount of finance deployed^5^.

The private sector plays a central role in adaptation and resilience (A&R) as providers of adaptation goods, services, innovation and investment to the broader economy. In adapting and building their own resilience, firms can also contribute to societal resilience by generating resilient services, jobs and growth. Conversely, by failing to adapt or implementing risk responses that undermine resilience for others, firms also risk pushing societies down maladaptive pathways^6^.

The financial sector is becoming increasingly interested in adaptation, evidenced by the numerous frameworks and taxonomies published by non-state actors to guide investment (see review^7^). Growing evidence shows that physical climate risks represent material financial risks, with current and expected impacts on asset values^8–10^ and probabilities of default of firms^11,12^. Financial institutions (FIs) struggle to access the information that they require from firms to manage these risks, particularly concerning actions being undertaken to adapt. This information can have decisive implications. To give an example, extreme floods (1-in-a-1000-year events) in Thailand are estimated to cause a sovereign credit rating downgrade of four notches. In comparison, the same models estimate only a downgrade of two notches when considering a potential adaptation scenario^13^. Relevant adaptation information can therefore mitigate the modelled impact of risks significantly. For this reason, governments also require similar, though not identical, information on how firms (and as a result, FIs) are adapting, which they can aggregate to industry- or economy-level.

Adaptation also represents a significant investment opportunity for FIs. FIs want to identify well-adapted firms that will outperform in their markets at future climate, either by adapting their own operations and assets or by providing adaptation products and services that will be increasingly needed to cope with climate change. The market for adaptation-related products and services is expected to be significant. Some estimates suggest a market investment opportunity of around $400 billion per year for climate adaptation alone^14^, others as high as $2 trillion per year^15^. Recent research by the London Stock Exchange Group^16^ suggested that the size could already be $1 trillion per year, with particular opportunities related to water, food and real-estate sectors.

One of the principal barriers to adaptation and to scaling adaptation financing cited is the dearth of reliable information^17^. Unlike with emissions, no detailed, quantified ‘adaptation and resilience trajectories’ disaggregated by sector and technology exist against which the ‘alignment’ of financial flows, companies or whole economies can be assessed. Indeed, arguably, despite recent progress on a Global Goal for Adaptation, it is unlikely that an adaptation equivalent of ‘net zero’ will exist. Instead, there will likely continue to be a patchwork of inconsistent qualitative goals specific to individual sectors or geographies. As a result, FIs and regulators rely on evaluating adherence to principles, such as managing environmental physical risk, doing no significant harm to society and nature, aligning with third-party adaptation plans, and contributing actively and positively to resilience^5^, as forms of measuring ‘alignment’ with societal A&R. This is evident in frameworks such as the EU Taxonomy, where adaptation-related metrics are process-based. Investors, banks and insurers need information on a company’s adoption of these principles for multiple uses: to price risk, inform client/investee engagement and stewardship strategies, design products, and make capital allocation decisions. Governments and regulators also need similar information to assess the preparedness of economies for environmental risks and appraise where intervention might be required to address the gaps. However, this data is missing—or so is argued.

The lack of decision-useful corporate sustainability data is not a new phenomenon. In fact, a series of disclosure frameworks and guidance, such as those by the Taskforce for Climate-Related Disclosures (TCFD), Taskforce for Nature-Related Disclosure (TNFD), International Standard-Setting Board’s (ISSB) IFRS S2 and Transition Plan Taskforce (TPT), have been published to encourage cohesive corporate disclosure of sustainability information. In some jurisdictions, these have even become mandatory (e.g., UK and EU). The ensuing disclosures have given rise to a series of assessments that evaluate the compliance and performance of firms with these frameworks^18,19^. Some have started assessing the quality of disclosures to counteract the risk of greenwashing^20,21^, while others have even started using disclosures to estimate firm-level risk^22–24^. It may therefore seem that the issue has been sufficiently addressed.

However, there are no dedicated A&R disclosure frameworks, which makes it impossible to assess firm compliance as a proxy for physical risk and alignment with A&R. Equally, while myriad methodologies exist to estimate firm-level exposure to climate risks (e.g., refs. ^25–27^), there is no authoritative way of integrating the adaptation measures companies have taken to address specific risks or their capacity to take these measures (i.e., a company’s adaptive capacity). Given that firms’ own adaptive actions are well known to be a dominant driver of risk, this means that most physical risk assessments in the literature to date are biased or incomplete when it comes to the performance of individual firms (see the example in ref. ^13^ for sovereigns). The focus on firm- or asset-level risk profiles in the literature also does not capture the full extent of contribution to and alignment with societal A&R. To date, FIs and regulators are thus reliant on corporate disclosures to evaluate corporate alignment with A&R principles.

Although the various taskforces guiding corporate disclosure have not explicitly centred A&R, there is some overlap. For example, 22.a in the ISSB’s IFRS 2 states that “[a]n entity shall disclose information that enables users of general-purpose financial reports to understand the resilience of the entity’s strategy and business model to climate-related changes, developments and uncertainties, taking into consideration the entity’s identified climate-related risks and opportunities”. The CDP questionnaire includes sections on climate adaptation actions taken, as well as freshwater use and land use^28^. The TPT has laid out broad principles for integrating adaptation into transition planning, and these have recently been elaborated further by many organisations, including the latest guidance published by the ISSB, the European Financial Reporting Advisory Group, and the Network for Greening the Financial System. There is, therefore, potential for sustainability disclosures to already include substantial A&R-related information.

Indeed, a rich academic literature exists utilising CDP disclosures to infer the extent to which corporates are planning or responding to climate change. Studies have variously found climate vulnerability, uncertainty, risk management capabilities, and awareness of climate-related risk to drive corporate adaptation^29–32^, particularly when viewed through an organisational theory lens. Others have been informed by environmental determinism, strategic choice and attentional views on corporate adaptation strategies to determine which stimuli have driven action^33–35^. Others have used CDP questionnaires to characterise adaptation planning blind spots^36^. These have been complemented by more recent studies looking at private investment using multiple unlisted data sources^37,38^. While foundational, none have explicitly considered the volume of A&R information that can be found in sustainability reports—strategic narrative reports that intend to encapsulate a broader set of non-financial information.

This study is the first to empirically analyse the extent to which information being disclosed by corporates in their sustainability reports includes information on A&R. Given the limitations of the information captured in sustainability reports^39,40^, as well as broader concerns around greenwashing and greenhushing, we only make light interpretations under the assumption that the information disclosed reflects firms’ adaptation responses. Instead, this paper serves to guide future use of sustainability reports, academic and beyond, as a source of A&R information. Furthermore, the methodology developed can be applied to other forms of disclosure (e.g., US filings or other financial reporting), which may be deemed more trustworthy. At the same time, the dataset itself can be utilised to delve deeper into the drivers and types of adaptation response in line with the literature outlined in the preceding paragraph.

We develop a 91-indicator assessment framework and use it to assess the latest sustainability reports of the S&P 500 companies with a large language model (LLM) to generate 42,030 datapoints for our analysis. In so doing, this study makes four contributions to the literature. First, the paper builds upon existing disclosure frameworks (i.e., TCFD, TNFD, ISSB IFRS S2 and the TPT) and literature (e.g., refs. ^5,41^.) to develop a common framework for assessing the extent of corporate A&R disclosure. Our assessment framework integrates with existing reporting standards and expands their A&R components. Noting the critical semantic and ontological differences between adaptation and resilience, this study generally adopts IPCC definitions but uses these terms in unison to avoid complex attributions of individual indicators to one concept over the other, and to reflect broader industry trends. We define adaptation and resilience to encompass both physical climate and nature-related risks, given the evidence of their interdependencies. A common framework is expected to enhance firm A&R reporting while making public information more comparable and usable for investors and regulators. Second, the paper develops and applies an LLM to assess the extent of A&R information reported by corporates, allowing interested parties (e.g., firms, investors, governments, regulators, CSOs, and researchers) to action their own assessments with ease. The benefit of making assessments more accessible is that they encourage adoption and further research. Third, the paper is the first to empirically validate claims that existing disclosure is insufficient to enhance A&R decision-making. In using the disclosure of S&P 500 companies as a proxy, the paper provides a snapshot of the disclosures of firms with a combined valuation of $43 trillion regarding adapting to climate change and building resilience^42^. Based on our findings, we create four archetypes of disclosers. We also consider companies targeted by the Climate Action 100+ (CA100 + ) and Nature Action 100 (NA100) initiatives separately, to examine in detail the current state of high-impact companies already committed to enhanced sustainability disclosures. Overall, we show that corporate A&R disclosures are patchy and lacking significantly across specific themes (e.g., targets and metrics).

## Results

### Analysis on aggregate

**Fig. 1:**
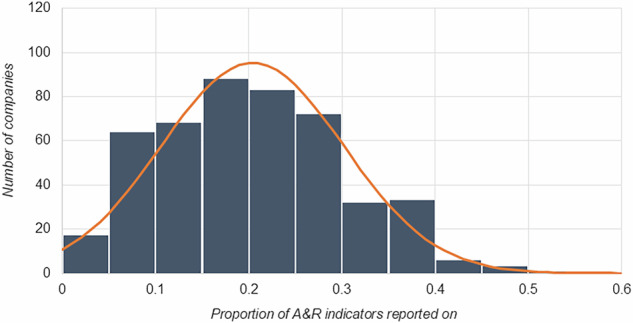
Normal distribution of the number of indicators reported on aggregate by S&P 500 companies. The x-axis demonstrates the percentage of A&R indicators the LLM determined companies report on. The y-axis demonstrates the number of companies at each percentage interval. The orange line represents the normal distribution.

Our analysis shows that on average, S&P 500 companies report against 20% of indicators in a comprehensive framework of corporate disclosure points (see Fig. 1) (refer to Methods and Supplementary Table 2–7 for details on the framework). Within this dataset, there is a high degree of variation in reporting, with the least reporting company providing information for only 1 of the 91 indicators, and the most-reporting company providing information for 50, which still only covers just over half of decision-useful A&R indicators. Companies part of the NA100 and CA100 + , on average, report on more indicators in the framework than other S&P 500 companies, though this variance is not statistically significant. There is high variability in the types of indicators companies report on. An overview of the indicators over half of the assessed companies report on, and that five or fewer companies report on, can be seen in Table 1 (the complete list of indicators and proportion of S&P500 companies reporting on these can be found in Supplementary Tables 2–6)

**Table 1:** Indicators for which information is disclosed by more than 50% and less than 1% of S&P 500 companies surveyed

ID	Disclosure element	Sub-element	Indicator metric	% Yes
Most				
71	Governance	Institutional governance mechanisms	Has the company assigned the responsibilities of assessing and managing climate- and nature-related issues to a management-level position or committee that reports to the board?	91%
46	Implementation	Operations	Does the company report any policies or conditions used to ensure no significant harm is done to societal resilience through its business activities?	85%
75	Governance	Institutional governance mechanisms	Does the company have a process in place to escalate any issues that may cause significant harm to climate, nature and society?	68%
45	Implementation	Operations	Does the company report any policies or conditions used to ensure no significant harm is done to nature or ecosystem services?	67%
18	Risk	Identifying climate risk	Does the company have a specific process in place to identify physical risks arising from climate change?	66%
57	Engagement	with value chain	Does the company report any current or anticipated changes to upstream sourcing practices and interactions with downstream partners to address physical nature-related issues? (e.g. adoption of improved tracing, certification practices, collaboration with suppliers, customers and other stakeholders, or extended producer responsibility schemes)	66%
35	Risk	Risk management process	Are the company’s processes for identifying, assessing, prioritising and monitoring physical climate- and nature-related risks integrated into its overall risk management process?	64%
73	Governance	Institutional governance mechanisms	Does the company have a mechanism for individuals and communities to raise complaints when they may be adversely affected by the company?	64%
56	Engagement	with value chain	Does the company report any current or anticipated changes to upstream sourcing practices and interactions with downstream partners to address physical climate-related risks and opportunities?	55%
74	Governance	Institutional governance mechanisms	Does the company have a policy of non-reprisal against complainants, including human rights defenders, whistle-blowers, and community spokespersons?	55%
66	Governance	Institutional governance mechanisms	Does the company report the processes used through which the board or board committees are informed about physical climate-related risks or opportunities?	51%
Least				
10	Foundation	Disclose physical nature risks	Does the company report the physical risks to its business operations arising from nature loss and ecosystem degeneration?	0%
42	Implementation	Operations	Does the company refer to external definitions or taxonomies to classify its products and services as resilience aligned?	0%
51	Implementation	Operations	Does the company report on the scenario tools and methodologies used to test the resilience of its financial business strategy on nature-related issues?	0%
90	Metrics & Targets	Metrics	Does the company report its climate adaptation-aligned capital expenditure?	0%
34	Risk	Identifying nature risk	Does the company report the frequency at which it carries out assessments of nature-related risks and opportunities?	0%
79	Governance	Links	Are the company’s performance metrics for nature-related dependencies, impacts, risks or opportunities included in its remuneration policies?	0%
86	Metrics & Targets	Targets	Are the company’s climate- and nature-related targets validated by an independent third party?	0%
24	Risk	Identifying climate risk	Does the company report whether fat-tail risks or tipping points were considered when identifying physical climate-related risks or opportunities?	0%
32	Risk	Identifying nature risk	Does the company report whether ecological thresholds or tipping points were considered when identifying nature-related dependencies, impacts, risks and opportunities?	0%
33	Risk	Identifying nature risk	Does the company report the frequency at which it carries out assessments of nature-related dependencies and impacts?	0%
65	Governance	Institutional governance mechanisms	Does the company report the number or proportion of board members with competence in nature-related issues?	0%
83	Metrics & Targets	Targets	Does the company provide an explanation of how its climate adaptation-related targets align with the Sharm-el-Sheikh Adaptation Agenda or other similar adaptation goals?	0%
91	Metrics & Targets	Metrics	Does the company report its nature-aligned capital expenditure?	0%

The most disclosed indicators include areas such as board oversight, social and environmental safeguards, and engagement with value chains. Notably, over half of the companies report having a process in place to assess climate-related physical risks—though less than 5% assess nature-related physical risks (ID26). Generally, these indicators represent areas that are easily implemented with minimal disruption (e.g. board oversight) and are broad enough for companies to report some information—i.e., they constitute low-hanging fruit. These indicators also encompass issues that companies have been encouraged to take action on as part of a longer-standing and broader sustainability agenda (e.g., DNSH, reducing environmental impact, etc.). On the other hand, the indicators S&P 500 companies report the least on relate to companies’ metrics and targets, nature-related financial risks, capital expenditure linked to adaptation or natural capital, and the precise mechanics of their risk assessments, particularly around uncertainty and non-linearity. Reporting against these indicators requires companies to engage in more depth with A&R, going beyond what is currently included in TCFD and other frameworks and make more significant adjustments to business practices. Moreover, the specificity of this set of indicators means that reporting this information requires implementation of best practice (e.g., using scenarios to assess the resilience of the business strategy). Importantly, the indicators in this list suggest a lack of commitment to nature-related issues beyond engaging with their value chain—66% of companies report some form of nature-related value chain engagement (ID57). Overall, these findings reflect a high degree of variance in corporate reporting on resilience indicators. Our analysis is therefore extended to consider what trends emerge when aggregating indicators across disclosure elements and sub-elements.

### Variation between disclosure elements and sub-elements

**Fig. 2:**
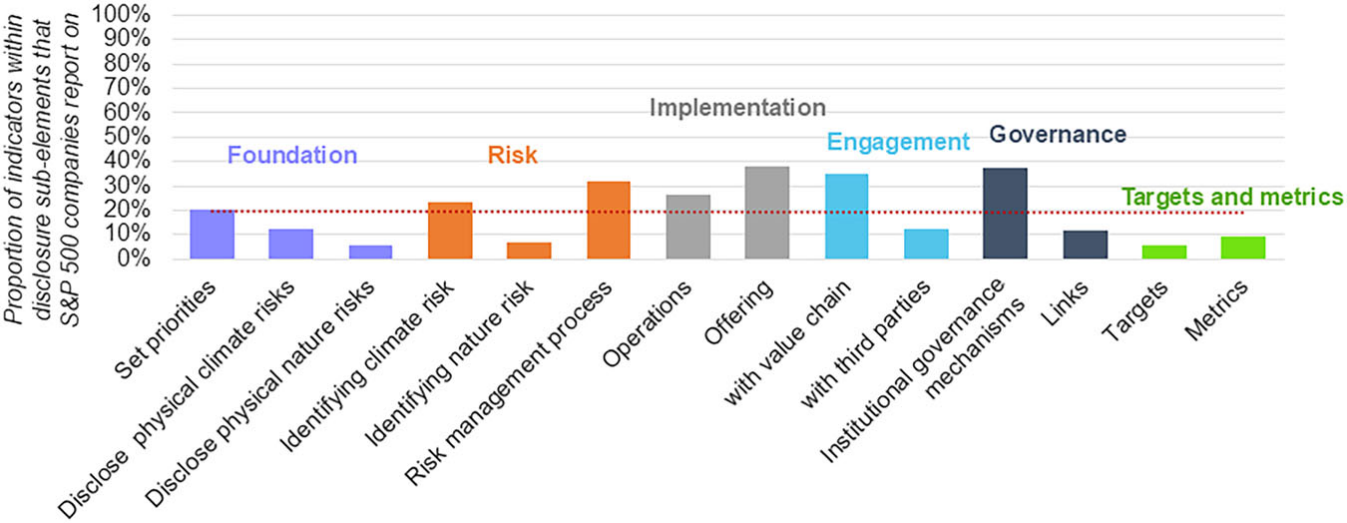
Proportion of indicators within a disclosure sub-element that S&P 500 companies report against on average. Indicators represent decision-useful A&R information points. The y-axis shows the percentage of indicators within a disclosure sub-element from our assessment framework that the LLM determined companies report on, averaged across all companies in our sample. The x-axis lays out the disclosure sub-elements of the assessment framework and groups these further into the underlying disclosure elements (distinguished by different shading). The red dotted line is the average proportion of indicators S&P 500 companies report on.

Some trends emerge when considering differences in indicators between each of the disclosure elements and sub-elements. *Metrics & Targets* indicators are reported on the least frequently by companies (7% of the time on average). The indicators in this element cover the types of metrics, targets, and links with remuneration packages companies have instituted for the management of climate- and nature-related risk and A&R solutions more broadly. Publicly reporting on their targets and metrics enables investors and supply chain partners to hold companies to account for their progress. Together with remuneration packages, these indicators can therefore serve as a measure of how committed a company is to addressing A&R. Though not final, companies will be hesitant to re-adjust their targets and metrics once they have been launched, which may explain the lack of targets, metrics and corresponding ties with remuneration packages companies have in place. Similarly, the S&P 500 companies report on only 11% of *Foundation* indicators, which means limited information can be found on how their business strategy aligns with resilience and the material risks and impacts they have identified. However, there is high variation within this disclosure element as companies, on average, report on four times more indicators related to their business strategy than the specific nature-related risks they are affected by (see Fig. 2 for a full breakdown across disclosure sub-elements).

Looking at the other end of the spectrum, on average, S&P 500 companies report against 32% of the indicators in the *Governance* category – the most across all disclosure elements. Within this element, 42% companies report on their board reporting mechanisms (i.e. ID68-70), 91% on their managerial oversight (i.e., ID71) and 53% on how safeguarding is ensured (i.e., ID72-75). While these indicators specifically question whether physical risks and adaptation have been incorporated into existing governance processes, they still constitute low-hanging fruit as they do not require significant changes – the ‘environment’ is recognised as a source of risk in conventional risk management and business processes already. However, while 50% of companies explicitly mention that climate-related risks and opportunities are reported to the board, only 3% report the same for nature. This distinction, again, is likely owed to the relative maturity of climate- and nature-related corporate disclosure frameworks. Moving beyond the low-hanging fruit, the analysis finds that only 3 and 0% of companies report whether their board have expertise on climate- (ID64) and nature-related issues (ID65), respectively. Equally, only 1% of companies report having developed a climate change adaptation plan (ID63). Around 19% of companies report having some initiatives in place to educate staff on climate- and nature-related risks (ID76-77), while 4% of companies report having embedded climate- and nature-related performance metrics into their remuneration policies (ID78-79). These indicators require dedicated resilience actions and therefore point to a lack of active engagement by corporates. Similarly, the relatively high degree of reporting from companies on *Implementation* indicators is skewed by companies’ more extended engagement with practices related to safeguarding, while areas requiring dedicated resilience actions, such as investments and asset disposals in response to climate- and nature-related risks (ID46-47), are not as widely reported (around 5% of companies on average).

The highest observed standard deviation between indicators in a disclosure element is found in *Engagement* indicators (see Supplementary Table 8 for a complete overview). Here, one company reports on 85% of the indicators while another reports on zero. This result suggests that the extent to which companies report against these indicators is more polarised—with multiple companies disclosing relevant information for nearly all indicators, while others do not report any. This could arise from established engagement opportunities not being present for specific companies, given their industry or national context, thus requiring a higher degree of initiative to seek opportunities out. Equally, in some industries such as Utilities, active engagement with the value chain or government may be more central to conventional business activities compared to others. Notwithstanding, S&P 500 companies are not significantly less or more likely to report information on *Engagement* than other disclosure elements.

### Variation between climate and nature

We also analysed whether there was variation in company reporting against paired climate-related and nature-related indicators—indicators that are generally the same except for considering climate- or nature-related factors. On average, companies are twice as likely to report on climate-related topics (20% of indicators) compared to nature-related topics (9% of indicators). These trends are observed more strongly when looking at paired nature- and climate-related topic indicators within disclosure elements. Companies describe 5x and 3x more often how physical climate risks are incorporated into their governance processes and how they are assessed than for physical nature-related risks. However, the differences in reporting on *Metrics & Targets* indicators for climate- and nature-related risks are minimal, while the results show that companies report a bit more frequently on engagement related to nature-related risks than climate (though not significantly). These findings suggest that physical climate-related risk disclosures are more mature than physical nature-related risks, in part due to the legacy of TCFD, which predates TNFD. While companies generally comply with ‘mainstream’ safeguards, some of the more recent considerations of nature-related risks and dependencies are still lacking.

### Variation between sectors

No significant variation emerges when segmenting the average scores of companies by GICS industry level. However, some trends can be identified when further splitting the GICS industry level averages across indicator elements. For example, Real Estate companies report on *Risk* indicators more often than other industries. In contrast, Consumer Staples, Utilities, and Materials companies report on *Engagement* indicators 2-3.5x times more often than Real Estate and Financial sector companies. This reflects industry characteristics, such as real estate being more exposed to climate-related physical risks, and Consumer Staples, Utilities and Materials companies taking part in more engagement initiatives either as part of engaging with consumers, value chains or governments. Furthermore, Consumer Staples companies report on 6x more *Metrics & Targets* indicators than Financial, Communications, and Industrial sector companies. This latter finding suggests that the Financial, Communications and Industrial industries are lagging when it comes to demonstrating accountability for their work on A&R.

**Fig. 3:**
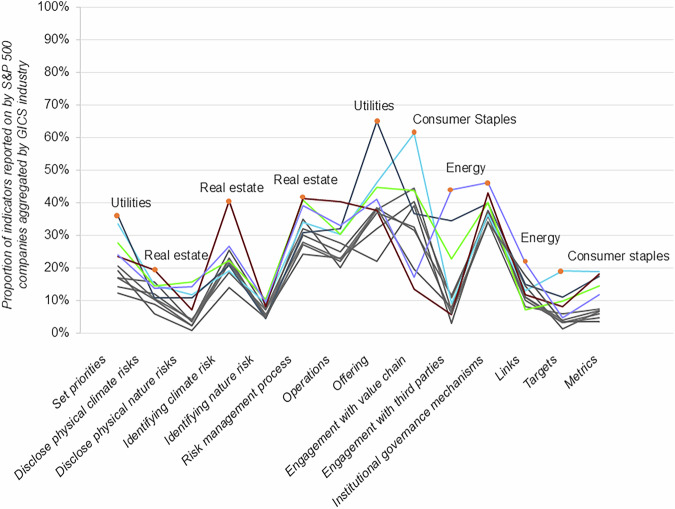
Proportion of indicators reported on by S&P 500 companies, aggregated by GICS industry and disclosure sub-element. Only those industries with significantly higher values are highlighted in a different colour. The orange dot signifies higher reporting rates compared to other industries, with a 95% confidence interval.

These results can be segmented further when examining variations between disclosure sub-elements (see Fig. 3). Notably, Real Estate companies perform relatively well on climate-related physical risk indicators and *Operations* indicators compared to other sectors. However, it should be noted that these companies still report on less than half of the indicators included (most of which focus on nature-related risks). Utilities companies report on more *Set Priorities* and *Offering* indicators than other sectors, likely due to the closer regulatory supervision and their product offering being an adaptation solution (e.g., water treatment). Similarly, Energy companies report more often on their *Engagement with third parties*, *Institutional Governance Mechanisms* and *Links* indicators than other sectors. This again can be attributed to companies engaging more frequently with governments through contractual arrangements and, therefore, requiring stronger safeguarding policies. Overall, these results indicate that some inter-industry variation exists in the type of information disclosed by different companies. In many of these cases, the differences reflect the type of product offered or the extent to which engaging with other actors or fortifying internal policies is a competitive requirement (e.g., governments, consumers). Interestingly, there are no industries that perform significantly worse than others on specific disclosure elements. This is likely due to the already low average score of companies across the dataset.

### Variation by cluster

**Fig. 4:**
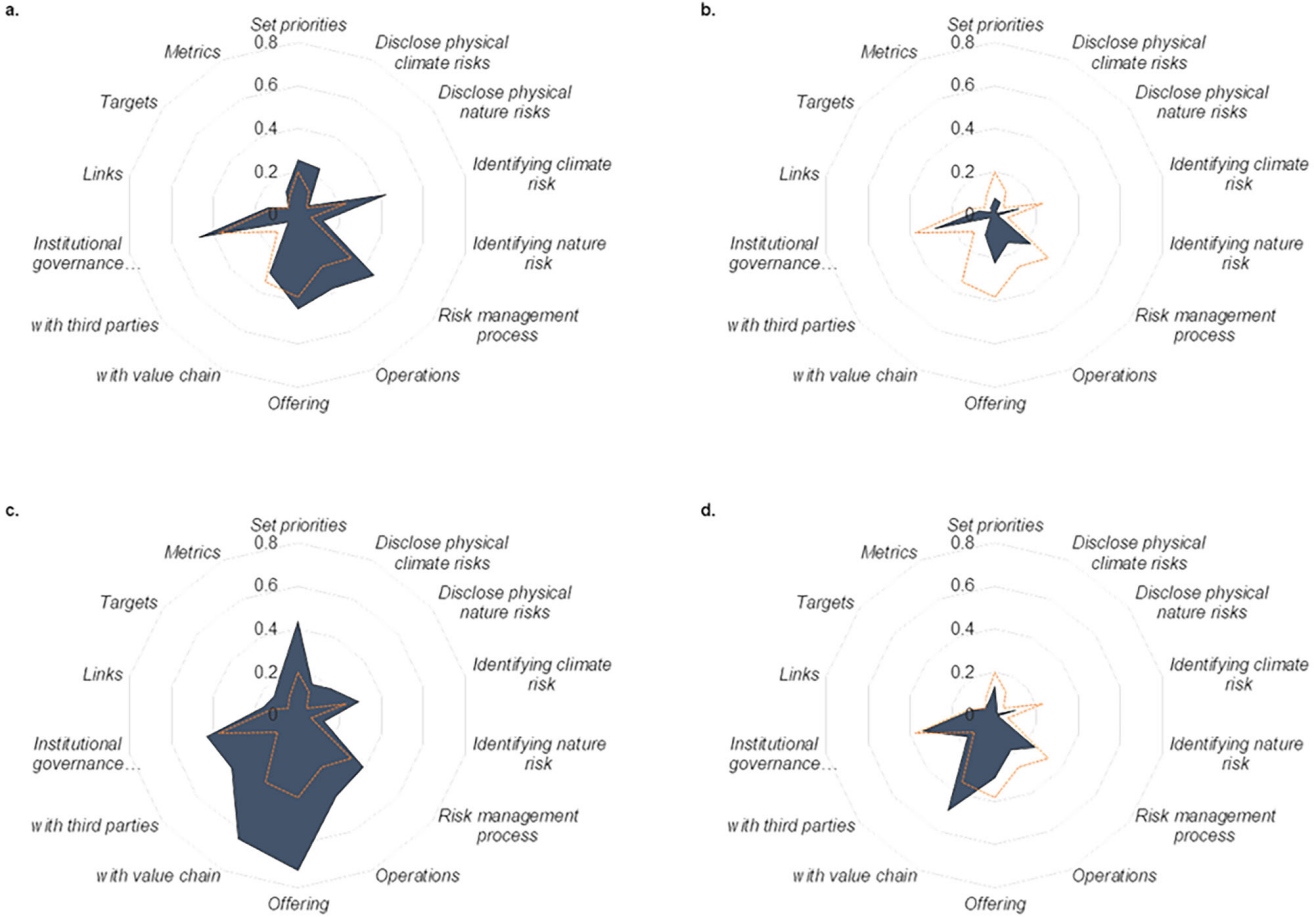
Types of discloser identified in k-means analysis. **a–d** Mean percentage of indicators reported on by different clusters, disaggregated by disclosure sub-element (represented in dark blue). The orange dotted line represents the percentage of indicators reported on by the sample average company. The clusters are composed of S&P 500 companies and were categorised using a k-means analysis. **a** Cluster A - ‘Climate Risk Aware’. **b** Cluster B - ‘Laggards’. **c** Cluster C - ‘ Product Aligned’. **d** Cluster D - ‘Engaged’.

**Fig. 5:**
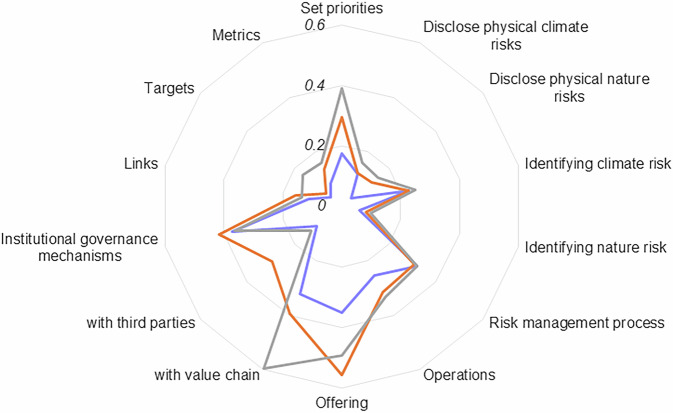
Average disclosures across indicator sub-categories, disaggregated by companies targeted by the CA100+ (orange) and NA100 (grey) initiatives, and those not targeted by these (purple).

**Fig. 6:**
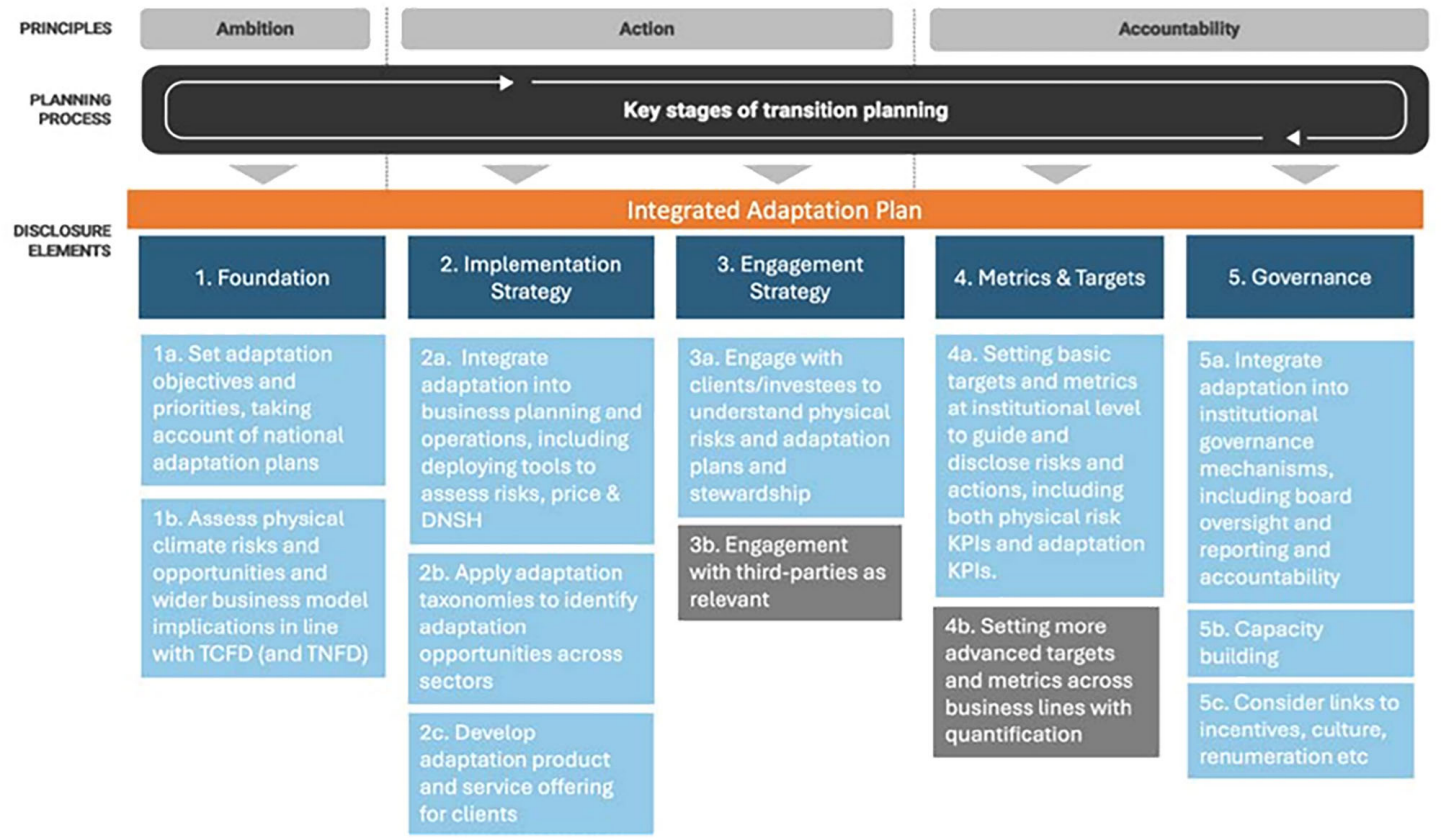
Integrated adaptation planning framework. Conceptual framework for adaptation planning, with 12 specific action areas (in light blue and grey, 1a to 5c) developed by the Oxford Resilient Planet Finance Lab with expert input from the CFRF Adaptation Working Group. ‘Grey’ action areas (3b and 4b) indicate ‘stretch’ actions, while those in blue are foundational. The top two rows (principles and planning process) and the 5 disclosure elements (in dark blue, labelled 1 to 5) exactly follow the TPT framework. The link to the transition planning process, as defined by the TPT, aims to demonstrate how ‘resilient transition plans’ or adaptation can be fully integrated within the transition planning cycle.

Given the large number of companies, a k-means cluster analysis was conducted on the mean number of indicators that companies report information on across different disclosure elements to identify any relationships between these. In running the analysis, four clusters emerged as the optimum number for k-means clustering (see Figs. 4–6). Cluster 1 covers 126 companies that report higher than average on *Risk*, *Foundation*, *Implementation* and *Governance* indicators, but lower than average on *Engagement* indicators. Cluster 2 comprises 132 companies that report lower-than-average performance across all indicators. Cluster 3 includes 140 companies that report on more Metrics & Targets, Foundation, Engagement, Implementation and Risk indicators (in that order)—all disclosure elements except *Governance*. On the other hand, Cluster 4 is comprised of 69 companies that report higher than average on *Engagement* indicators and lower than average on *Metrics & Targets*, *Implementation*, *Risk* and *Foundation* indicators.

Figure 4a–d shows the profiles of the four clusters of companies identified. The average number of indicators reported on by companies in each of the clusters is 27%, 11%, 32%, and 16%, respectively. The means across disclosure elements hide the variation between clusters, due to the high variation between the indicators within a disclosure element (as seen in Fig. 4a–d. However, when examining the disclosure sub-elements, specific characteristics emerge that help profile the clusters. Companies in Cluster 1 report a relatively high degree of information on climate risk, risk management processes, and institutional governance mechanisms, as well as some efforts to strengthen their operations and offering. These characteristics suggest that companies have already taken up some of the recommendations from TCFD, for example, or have started mainstreaming climate-related risk management into their operations, without necessarily explicitly addressing A&R (hence ‘Climate Risk Aware’). Companies in Cluster 2 generally do not report on A&R indicators, although there is some information related to *Governance* indicators (hence ‘Laggards’). Cluster 3 companies are the relative high-performers of the dataset, often reporting on indicators related to their *Offering*, *Value chain engagement* and *Business priorities*. However, they report less information on *Risk* indicators compared to the ‘Climate Risk Aware’ companies. Without explicitly considering physical climate- and nature-related risks, companies are likely scoring highly on A&R due to overlap with existing business practices (hence ‘Product Aligned’). On the other hand, Cluster 4 companies are low performers whose A&R-related reporting has focused on value chain engagements. This suggests that some companies may have been engaged on A&R issues through their value chain but are yet to incorporate these into their own business practices (hence the ‘Engaged’). Similarly, focusing on engagement activities without advancing A&R in other areas of their business may be seen as an attempt to greenwash.

The cluster profiles are corroborated by examining industry composition of the clusters. Over a third of the Utilities, Materials and Consumer Staples companies in the S&P 500 are part of the ‘Product Aligned’, while only 6% of IT and 7% of Real Estate companies are part of this cluster. Interestingly, only 2% of Healthcare companies are part of this group, suggesting that companies in this industry could benefit from explicitly aligning their reporting with A&R. 60% of the Real Estate companies in the S&P 500 are in the ‘Climate Risk Aware’ cluster, reflecting the industry’s high-level exposure to climate-related physical risks. Just under half of the Communications Services and Financial companies of the S&P 500 fall into the ‘Laggards’ cluster, while between 34% and 39% of IT, Consumer Discretionary, Healthcare, and Industrials companies fall into the ‘Engaged’ cluster. This again reaffirms the potential for healthcare companies to align with A&R, given that this is an industry continuously identified as an adaptation priority^7^. Overall, the results of the cluster analysis align with the descriptive analysis in the preceding section.

### Variation by other factors

When segmenting the sample of S&P 500 companies by membership of the CA100+ and NA100 initiatives, differences emerge (see Fig. 5), which vary in significance and effect size (see Table 2). Across all indicators, companies identified by either initiative report more A&R-related information on average than companies not targeted by these initiatives. The effect is most pronounced across *Foundation* and *Implementation* indicators, where both CA100+ and NA100 companies are more likely to report A&R information. The NA100 companies report particularly more information related to *Foundation*, *Engagement* and *Metrics & Targets* indicators than other S&P 500 companies. By contrast, reporting does not differ significantly for *Risk* and *Governance* indicators when comparing NA100 and CA100+ companies with other S&P 500 companies. We find no significant correlation between firm market value and proportion of indicators reported on in our sample, suggesting that the effect of NA100 and CA100+ membership is not simply a result of firm size. Beyond the slightly higher levels of disclosure though, companies targeted by neither initiative report on materially different disclosure elements compared to other companies part of the S&P 500.

**Table 2:** p-value (statistical significance) and d-value (effect size) for the variance in average disclosure of firms part of the NA100 or CA100+ initiatives compared to S&P500 companies not identified in these initiatives, disaggregated by indicator category

*Variance with other S&P 500*	*All indicators*		*Foundation*		*Risk*		*Implementation*		*Engagement*		*Governance*		*Metrics and targets*	
	NA100	CA100+	NA100	CA100+	NA100	CA100+	NA100	CA100+	NA100	CA100+	NA100	CA100+	NA100	CA100+
*p-valu*e	3E-05	2E-04	3E-06	2E-03	4E-01	8E-01	2E-04	3E-05	1E-06	8E-04	9E-01	5E-02	8E-05	8E-02
*d-value*	0.75	0.60	0.98	0.59	0.16	0.05	0.63	0.66	0.94	0.62	-0.01	0.33	0.85	0.31

(continues overleaf).

We also control for the impact of recent ‘green’ innovation activity (as in^43^) on the proportion of A&R indicator reporting and find no significant relationship, even when narrowing the analysis to *Implementation* and *Offering* indicators. This suggests that the limited ‘green’ patenting activity that has taken place does not explicitly address A&R issues or is not reported as such.

## Discussion

The fundamental contribution of this paper is to empirically validate the claim that existing corporate sustainability reports do not include sufficient information to enhance A&R-related decision-making by FIs and regulators. Our analysis of 42,030 data points shows that surveyed S&P 500 companies report information related to only 20% of a comprehensive set of A&R indicators built on existing disclosure frameworks, best practice and expert input. This percentage is lower than the 36% of decarbonisation-specific indicators reported on by CA100+ companies^21^. The analysis has demonstrated some (though still limited) disclosure in line with existing frameworks, with, for example, half of the companies surveyed providing information regarding their climate risk assessments (ID18), in line with TCFD. However, limited reporting persists for indicators beyond existing frameworks. This finding adds weight to the importance of dedicated A&R frameworks to secure comprehensive corporate disclosure. Within the sample of companies, important variations are found. For example, on average companies report on less than 10% of indicators related to metrics, targets, and nature risk, while they report on nearly 40% of indicators related to the governance mechanisms they have introduced and how their product offering is aligned with A&R. In reviewing the most and least commonly disclosed indicators, it becomes clear that companies’ average disclosure percentage is being boosted by low-hanging fruit indicators that either require slight adjustment to current business operations, are broadly formulated, or have a longer history on mainstream sustainability agendas. These findings suggest corporate engagement with A&R has been limited to date, underscored by improper risk assessments that underappreciate risk and a reluctance to be transparent about the adaptation measures planned and taken to address these (if at all).

In line with the latter, our findings also show that companies are twice as likely to report climate-related information as equivalent nature-related information, reflecting the relative maturities of the TCFD and the TNFD disclosure frameworks – this lends credence to the effectiveness of established disclosure frameworks. Current nature-related disclosures focus on environmental safeguarding but overlook the impact of nature loss on businesses through dependencies and associated risks. Even without taking information disclosure as a proxy for the planning and actions taken by firms, the overall lack of information (and potential inaction of parties) on nature-related risks gives credence to the dangers of a green scorpion event materialising^44^. This finding is important to regulators and governments, who may therefore face mounting systemic risk across the economy and financial sector.

The comprehensive 91-indicator framework developed as part of this study captures information used to infer adaptation progress in multiple other studies. Differences in results emerge when comparing these. One study^31^, using CDP data, finds that 90% of companies identify climate-related opportunities, although our results based on sustainability reports suggest that only around 2% have identified opportunities. Equally, another^36^ also uses CDP data and finds that 76% companies report being impacted by climate-related physical risks, while only 40% of companies in our sample disclose this in their sustainability reports. The 2024 S&P Global Corporate Sustainability Assessment^5^ reports that 35% of companies have a physical risk adaptation plan, while our findings show that less than 2% of companies mention adaptation plans in their sustainability reports. Further research should explore what drives these differences: whether they arise from differences in population samples (e.g., the S&P study comprises a larger set of companies), methodologies (e.g., LLM-based over manual content analysis) or sources (e.g., sustainability reports, CDP questionnaires). These studies could provide further clarity on appropriate combinations.

The second contribution of this paper is to provide a snapshot of the trends in corporate A&R disclosure. Our initial analysis finds that specific industries, on average, report more on certain types of indicators. For example, Real Estate companies report more on risk-related indicators. Utilities companies report more information related to aligning with A&R through their business priorities and their product, which hints at the success of closer regulatory supervision in encouraging firms to address A&R. To explore this trend deeper, we construct four stylised types of disclosers (the ‘Climate Risk Aware’, ‘Laggards’, ‘Product Aligned’ and ‘Engaged’). The ‘Climate Risk Aware’ and ‘Product Aligned’ report more A&R-related information, likely due to higher exposure to climate-related physical risks and their product offering enabling A&R outcomes in the first place. However, both still fail to disclose information consistently on other A&R indicators, such as *Risk* and *Metrics & Targets*, which betrays a thorough engagement with A&R, given that risk is considered a foundational principle of A&R, and the lack of targets limits credibility. Industries are not uniformly represented in one of the discloser types, and there are notable exceptions of industries, such as Healthcare, that fail to position themselves with A&R despite their aligned product offering.

The inconclusive findings around inter-industry variation in the volume of A&R information disclosed in sustainability reports (including in our stylised discloser types) align with other studies capturing inter-industry variation within A&R more broadly. It is important to note that such empirical studies have predominantly focused on specific industries (as catalogued in ref. ^34^, or see ref. ^45^ for a recent example) and only a few incorporate a cross-industry angle. One study analysing private investment has found Manufacturing and Wholesale Trade to invest the least in climate adaptation relative to their gross value added, while Mining, Water supply, Administration and Defence invest the most^37^. Another^38^ analysing private sector investment related to floods in five coastal regions globally finds that Private services, Construction, and Industrial companies lag behind other industries in investment volume. Elsewhere, S&P^5^ find that Utilities and Real Estate companies are most likely to have an adaptation plan, while IT, Financials, Consumer discretionary, Communication services and Healthcare companies are, on average, less likely to have one. Patterns of corporate A&R disclosure in sustainability reports, other than the lack of A&R information disclosed by Healthcare companies, therefore, do not follow other A&R trends, and further research is needed to explore the cause of this.

When comparing S&P 500 companies targeted by the CA100+ and NA100 initiatives and those that are not, a significant positive correlation is found in membership and the level of A&R information reported. While it is not possible here to establish whether this relationship is causal, it is nonetheless a promising finding that the companies considered to have the most outsized impact on climate and nature are already reporting more A&R-related information. However, the results show that these companies simply report more A&R information within the same disclosure elements (e.g., *Governance*, *Engagement*, *Implementation*), while still not reporting on the types of indicators not reported on by the rest of the group (i.e., *Risk* and *Metrics & Targets*).

The implications of these findings for the literature are clear. Over the past four years, there has been a series of methodological innovations to estimate climate-related risk at the firm-level using a variety of novel techniques and data sources, including news, earnings calls, past Form 8-K issuances, and, crucially, sustainability reports. Missing across all methodologies are explicit considerations of A&R actions that may restrict a firm’s exposure and vulnerability. Our analysis finds that sustainability reports alone are grossly insufficient in providing the information necessary to incorporate A&R dimensions into these assessments, particularly considering how little *Implementation* and *Risk* information is being reported. Existing studies have often drawn on CDP disclosures, but other datasets, such as those related to investment, promise complementary data sources. Any future methodologies that attempt to holistically assess the extent to which firms are contributing to societal A&R will need to resort to other data sources to complement their analysis. Equally, further analysis is required to match specific A&R information points with stakeholder types and needs.

For literature examining the drivers, motivations or intentions underlying corporate adaptation, the disparities in our results with those based on CDP questionnaires suggest the methodology and database produced as part of this study may offer ground for complementary research. Information associated with some of the indicators in our framework could be bundled up to match existing conceptual frameworks (e.g. ref. ^32^) to derive insights on corporate risk management capabilities, awareness, and management of uncertainty. Further research in this vein could also explore how reliably disclosures can be used to proxy for corporate adaptation actions by ground-truthing disclosure levels with real-world risk events.

There are three main limitations to our findings and contribution. First, the analysis provided is binary and quantitative. Within reporting on A&R issues, there is, of course, scope for significant variations within indicators, for example, in the types of targets that are set or in how regular risk assessments are carried out. Reporting on a lot of the indicators in this framework, therefore, does not constitute actual contributions to A&R. Second, the assessment framework developed is sector-agnostic and thus focuses only on process-based indicators. Sector-specific indicators are needed for a more comprehensive evaluation of corporate A&R disclosure and performance, especially in the Implementation and Engagement categories, where, for example, indicators could analyse company water and land use. Third, corporates may implement A&R best practices without disclosing the full details of these in their sustainability reports. The LLM was found to be ‘stricter’ than the human assessor during validation, in that human assessors were more likely to consider the existence of information in other sources referenced (e.g. published corporate policies), which may distort the findings of the second contribution slightly. Future work will need to consider what metrics and datasets can be developed to accurately assess corporate A&R performance at the output or outcome level. Other work can consider how representative indices can be constructed hand-in-hand with prospective users based on this information, to give weight to certain information points over others and thus create effective at-a-glance evaluations.

The underlying contribution of this paper is the assessment framework used to analyse the extent of A&R-related information being disclosed and the LLM used to automate the analysis. We develop a framework of 91 binary indicators representing an attempt to exhaustively list all decision-useful A&R information points, drawing and expanding on the latest guidance from TCFD, TNFD and the TPT Adaptation Working Group (AWG). To our knowledge, the framework is the first detailed, dedicated A&R corporate disclosure framework. As such, it can guide firms, FIs and governments alike on the type of disclosure needed to enhance their respective decision-making.

## Methods

### The assessment framework

The assessment framework presented in this paper draws on the conceptual framework for corporate adaptation planning developed by the University of Oxford Resilient Planet Finance Lab with expert input from the UK Climate Financial Risk Forum’s (CFRF) AWG through a series of consultations in early 2024^46^. The CFRF AWG included 49 experts from FIs (insurers, asset managers, asset owners, private equity, banks), government, regulators, industry bodies, professional services, data companies, the UK Met Office, and research. The assessment framework builds on existing frameworks, such as the TPT, the ISSB IFRS2, TCFD and TNFD, but combines this with the climate resilience alignment framework of Mullan and Ranger^5^ and greater detail on targets and metrics from UNEPFI^41,47^, as well as expert inputs from the CFRF AWG to provide 12 specific areas of action. The Mullan and Ranger paper has been selected because its principles have been used to define alignment with adaptation and resilience in jurisdictional adaptation taxonomies^7^. Our assessment framework also explicitly responds to the call for integrated climate- and nature-related risk assessments and the synergies between nature and adaptation actions, including important insights derived from TNFD. The conceptual framework is given in Fig. 6 (see also ref. ^5^*)*.

The above conceptual framework presents best practice in corporate adaptation planning. It was assumed that information demonstrating adoption of or compliance with best practice would constitute effective A&R disclosure. We fleshed out the conceptual framework in Fig. 6 by harvesting metrics from 25+ disclosure frameworks focused on general sustainability-related disclosures, evaluating resilience contributions and categorising adaptation alignment (see Supplementary Table 1 for a full list). This resulted in 300+ unique A&R indicators grouped into the disclosure elements shown in Fig. 6. We teased out risk-related indicators into their own separate category, given their quantity and the importance of risk for A&R assessments. The resulting six disclosure elements of our assessment framework, therefore are: Foundation, *Risk*, Implementation, Engagement, Governance, and Metrics & Targets (see Table 3 for an overview). Upon compiling and categorising all relevant unique indicators, the list totalled over 300. This complete list of indicators was whittled down by focusing only on industry-agnostic and process-based indicators (rather than output- or outcome-based indicators, see^48,49^ for distinction). The former ensures the widest possible application of the assessment, while the latter ensures that relevant information can be found in sustainability reports. In this definition of indicators, we also referred to established A&R principles^5^ adopted by UNEPFI^47^, such as effective risk management, environmental and social safeguards, alignment with external adaptation plans, and robust monitoring processes.

**Table 3:** Overview of the indicator categories and sub-categories used to structure the analysis in this paper, with a short description of each

Disclosure element	Description
Foundation	
Set priorities	The company has clearly stated its ambition with regards to resilience, and this is reflected in its priorities and objectives.
Disclose physical climate risks	The company has disclosed its physical climate risks and opportunities and their locations.
Disclose physical nature risks	The company has disclosed its physical nature-related risks, dependencies and impacts and their locations.
Risk	
Identifying climate risk	The company explains the process it uses to identify physical climate risks and opportunities.
Identifying nature risk	The company explains the process it uses to identify its physical nature-related risks, opportunities, dependencies and impacts.
Risk management process	The company explains the processes it uses to manage its climate- and nature-related risks.
Implementation	
Operations	The company has implemented resilience in its business operations and planning processes.
Offering	The company has implemented resilience in its product and service offering.
Engagement	
with value chain	The company engages with its value chain to foster resilience.
with third parties	The company engages with third parties to foster resilience.
Governance	
Institutional governance mechanisms	The company has integrated resilience across its reporting and governance mechanisms, including the board and management, to institutionalise accountability.
Links	The company has taken measures to encourage resilience across its business practices.
Metrics & Targets	
Targets	The company has set targets to support resilience across climate and nature systems.
Metrics	The company uses relevant metrics to monitor its resilience.

To make these indicators implementable in the context of analysing company reporting through an LLM, they were turned into specific questions. Questions based on the indicators were draughted in a close-ended binary format to assess thoroughness of corporate adaptation reporting, following methodologies developed by Ni et al.^5^ and Colesanti Senni et al.^43^. As such, the questions necessarily provide a quantitative snapshot of the types of information being disclosed by firms and not a qualitative assessment of it. The resulting proposed assessment framework spans 91 questions across six categories (see Supplementary Tables 2–7 for the full framework).

### Sample and raw data

The companies part of the S&P 500 benchmark index were selected, as they give a wide coverage of sectors and represent around 80% of US market capitalisation with a combined valuation of US$43 trillion^50^.

The core data are the most recent sustainability reports published by companies, which were downloaded from their respective websites. The market value data used to control for firm size in the NA100 and CA100+ analysis was obtained from Compustat. The patent data used in that same analysis stems from Leippold & Yu^21^.

### Retrieval Augmented Generation (RAG)

The RAG system we use is based on the LlamaIndex open-source package (see ref. ^51^). To retrieve the relevant text passages for a question, we first split the underlying company report into chunks of a size of approximately 400 words. We allow a chunk overlap of 50 words to mitigate the loss of context due to random chunking. We use the sentencesplitter() function to obtain full-sentence form chunks^5^. To enable an effective search process, we need to transform chunks and search questions into numeric representations, the so-called text embeddings. This allows us to find semantically similar text chunks to the question. We rely on the OpenAI embedding model “text-embedding-ada-002” to transform the chunks and questions into embeddings. For each question, we retrieve the top 10 most relevant text chunks to answer a question. We use the state-of-the-art OpenAI LLM “gpt-4o-2024-05-13” to answer the questions and restrict the answer length to 200 words. For details on the prompt given to the model, see Supplementary Note 1.

The dataset employed for the illustrative use case encompasses the latest published sustainability reports from S&P 500 companies. Reports were procured for 476 companies. These reports were subsequently processed through the RAG model, which evaluated whether the provided information was adequate to address the indicator questions. The model’s output comprised a binary yes/no response, accompanied by an open-text justification derived from the extracted information from the reports. Supplemental directives were added for all (91) indicator questions to exclude certain information points based on expert domain-based knowledge (e.g., emissions reductions, etc) (refer to Supplementary Tables 2–7 for the complete list of indicators and their definitions).

Beyond the comprehensive testing and validation exercises conducted for this model, following the procedure of Schimanski et al.^52^. we also verified the accuracy of the tool’s outputs by hand. For this, an expert human analyst evaluated 455 indicators. The expert manually responded to a question based on the reports and compared the answer with the output of the LLM. Through this procedure, we identified that 83% of the LLM responses were correct. Discrepancies between the LLM and the human evaluator arose primarily from the human evaluator inferring additional information found in other publicly available sources referenced (e.g., a specific human rights policy), while the LLM relied more strictly on the information provided in the sustainability report. The LLM deviated most frequently on *Implementation* indicators, giving both false positives and false negatives when determining whether product offerings and corporate operations were aligned with A&R. This area likely requires enhanced directives or thresholds to determine alignment with more accuracy. It also underlines how vague existing information on *Implementation* indicators is.

### Statistical analyses

Post-model execution, averages were computed for each pairing within the analysis framework (i.e., companies, categories, industries, climate/nature indicators). Statistical techniques predominantly focused on assessing normality of data sources, identifying outliers, and using parametric and non-parametric evaluations of variance to determine significant differences.

### K-means clustering

K-means clustering uses an unsupervised machine-learning algorithm to create groups of data points with similar characteristics. The groups are created iteratively while optimising for the shortest Euclidean distance to the group centroid for each of its constituent data points. The data used was the average proportion of indicators per disclosure element reported on by each individual company. The elbow method was used to identify that 4 was the optimal value for k.

## Supplementary information

Supplementary Information

## Data Availability

The data we used for this study are public sustainability reports published by companies. This, together with the data produced by our tool, can be accessed at: https://github.com/robertospacey/A3F_SP500/.

## Code availability

The code used the develop the model on which the findings of this paper are based on, can be accessed via the accompanying paper here: https://arxiv.org/abs/2406.09818.
