# Supplementary material for: Empirically assessing corporate adaptation and resilience disclosure using AI
Source: NPJ Clim Action. 2026 Feb 12;5(1):22. doi: 10.1038/s44168-025-00321-7 (PMC12900641; doi:10.1038/s44168-025-00321-7)
Supplement: Supplementary file 1 — Supplementary Information [file 44168_2025_321_MOESM1_ESM.pdf]

Supplementary Table 1

Notes: To ensure complementarity and integration with existing frameworks, indicators from various frameworks were compiled and synthesised to inform the Adaptation Alignment Assessment Framework. The list of frameworks synthesised includes disclosure standards, performance measurement frameworks and taxonomies.

Supplementary Table 1. Full list of frameworks considered for indicator design.

Framework	Link
UNEP FI Physically Fit Report	link
ISSB IFRS S2 Climate-related Disclosures	link
IIGCC Working towards a climate resilience investment framework	link
CFRF Climate Disclosures Dashboard 2.0	link
SASB Industry Standards	link
TCFD 2021 Report, Appendix 2	link
TCFD Metrics & Targets Workshop Slides	link
IADB A Framework and Principles for Climate Resilience Metrics in Financing Operations	link
World Bank Resilience Rating System: A Methodology for Building and Tracking Resilience to Climate Change	link
GCA Adaptation Metrics Current Landscape and Evolving Practices	link
GBP Impact Reporting Working Group: Suggesting Impact Reporting Metrics for Climate Change Adaptation Projects	link
OECD DAC Rio Markers for Climate	link
GRI Reporting Standards	link
OECD Climate Resilient Finance and Investment	link
EBRD GET Technical Guide	link
Joint MDB Assessment Framework for Paris Alignment for Direct Investment Operations	link
Race to Resilience	link
Adaptation Solutions Taxonomy	link
FAST-Infra Sustainable Infrastructure Label	link
ACT Physical Risks & Adaptation	link
CDP Climate Change 2023 Scoring Methodology	link
IRIS+ System | Standards	link
Adaptation Resilience Impact Collaboration (ARIC): A measurement framework for investors	link

Equator Principles	link
EIB - MDBs Joint Methodology for Climate Finance	link
SBTN Technical Guidance: Step 1 - Assess	link
SBTN Technical Guidance: Step 3: Freshwater - Measure, Set & Disclose	link
Green Bond Principles / ICMA - Harmonised Framework for Impact Reporting	link

Supplementary Tables 2-7

Notes: The tables in Supplementary Table 2-7 show each of the indicator questions in the Adaptation Alignment Assessment Framework for each disclosure element. "ID" refers to the identifying number for the indicator. "Question" demarks the question developed for each indicator. "Definitions" covers supplementary information provided to the large language model used in the analysis to enhance the generated answers. The disclosure sub-elements are marked out in grey, with corresponding indicator questions appearing below these.

[tables made available overleaf]

Supplementary Table 2. List of indicators for *Foundation*.

ID	*Question*	*Definition*
**Set priorities**		
1	Does the company explicitly outline adapting to climate change as a priority in its business strategy?	Answer "Yes" only if the company prioritises either reducing its exposure to climate-related hazards (e.g. storms, floods, etc.) or enabling others to reduce their exposure to climate-related hazards. This statement can be general and not necessarily tied to a company's sector or industry. Do not consider any information related to emission reductions, decarbonisation or greenhouse gases.
2	Does the company commit to avoid and reduce its contributions to key drivers of nature loss and/or restore and regenerate ecosystems?	Key drivers of nature loss are broad processes that can lead to nature loss, such as land use change, overexploitation of resources, pollution or invasive species, among others. Do not include any information related to mitigating climate change or reducing greenhouse gas emissions. Answer "Yes" only if the company acknowledges through which drivers it contributes to nature loss and it commits to reducing its contribution to these drivers. Answer "No" if the company takes broadly about reducing environmental impacts.
3	Does the company reference any external laws, national policies or voluntary commitments relating to climate change adaptation in its business strategy?	Climate change adaptation refers either to reducing exposure to physical climate hazards (e.g. floods, droughts, etc.) or to enabling others to reduce their exposure to physical climate-related risks. Do not include any external laws, national policies or voluntary commitment related to reducing greenhouse gas emissions. Answer "Yes" only if the company outlines how specific parts of its business strategy either align or respond to priorities and commitments set in international-, national- or sector-based climate adaptation plans or laws. Answer "No" if the company only broadly talks about third-party adaptation plans or priorities without linking specific elements of its business strategy to these.
4	Does the company reference any external laws, national policies or voluntary commitments relating to nature loss and/or ecosystem restoration in its business strategy?	Nature loss broadly refers to the significant loss of ecosystem functioning, loss of biodiversity or loss of species abundance. Ecosystem restoration broadly encompasses activities seeking to strengthen 'provisioning' and 'regulating' ecosystem services. Do not include any external laws, national policies or voluntary commitment related to reducing greenhouse gas emissions. Answer "Yes" only if the company outlines how specific parts of its business strategy either align or respond to priorities and commitments set in national- or sector-based nature positive or biodiversity plans and laws. Answer "No" if the company only broadly talks about third-party nature positive or biodiversity plans or priorities without linking specific elements of its business strategy to these.
**Disclose physical climate risks**		
5	Does the company report the physical risks to its business operations arising from climate change?	Physical climate-related risks are a function of climate-related hazards, such as floods, the exposure of assets and operations to these hazards, and the vulnerability of these operations and assets of being disrupted. Business operations include a company's assets or activities. The risks can extend beyond the company to its value chain. Do not include anything related to climate change mitigation or reducing greenhouse gases. Do not rephrase of the physical climate-related risks mentioned by the company, instead use these in your answer. Answer "Yes" only if the

		company explicitly defines physical climate-related risks and explains how its business operations would be affected by these physical climate-related risks. Answer "No" if the company only broadly outlines different physical climate-related hazards or if the company identifies risks without clearly explaining how their business operations are affected by these.
6	Does the company report on its business opportunities arising from climate change?	Physical climate-related opportunities are a function of climate-related hazards, such as floods, the exposure of assets and operations to these hazards, and the vulnerability of these operations and assets of being disrupted. Business operations include a company's assets or activities. Responses to physical climate-related risks may result in new business opportunities for the company. New business opportunities include setting up new revenue streams, strengthening existing business operations or reducing costs of existing operations. The opportunities can extend beyond the company to its value chain. Do not include anything related to climate change mitigation or reducing greenhouse gases. Do not rephrase the physical climate-related opportunities mentioned by the company, instead use these in your answer. Answer "Yes" only if the company explicitly defines physical climate-related opportunities and explains upcoming or current plans to seize these. Answer "No" if the company only broadly outlines different opportunities from climate change without clearly explaining how they will change business operations to seize these.
7	Does the company disclose the location of its business activities affected by physical climate risks?	Physical climate-related risks are a function of climate-related hazards, such as floods, the exposure of assets and operations to these hazards, and the vulnerability of these operations and assets of being disrupted. Business activities include any operating, financing or investing activities. The locations may be disclosed in a map format or a list format. If you find a list of business operations affected by physical climate-related risks with geographical information for the business operations, then answer "Yes". If the company states that it discloses the locations of business activities affected by physical climate-related risks, then answer "Yes". Answer "No" if the company outlines locations or regions affected by particular physical climate-related risks without identifying its business operations affected by these.
8	Does the company disclose the locations of its assets affected by physical climate risks?	Physical climate-related risks are a function of climate-related hazards, such as floods, the exposure of assets and operations to these hazards, and the vulnerability of these operations and assets of being disrupted. Assets here includes any tangible assets used by the company (e.g. factories, farmland, etc.). The locations may be disclosed in a map format or a list format. If you find a list of company assets affected by physical climate-related risks with geographical information for the business operations, then answer "Yes". If the company states that it discloses the locations of its assets affected by physical climate-related risks, then answer "Yes". Answer "No" if the company outlines locations or regions affected by particular physical climate-related risks without identifying its assets affected by these.
9	Does the company identify the anticipated effects of physical climate-related risks on revenues, expenses, cashflows, asset and liability	Answer "Yes" only if the company provides an explanation of the anticipated effects across two or more timescales (i.e. short-, medium or long-term). Answer "No" if the company only explains the anticipated effects in one timescale, i.e. only short-term effects, only medium-term effects, only long-term effects.

	values or funding sources over the short- medium- and long-term?	
**Disclose physical nature risks**		
10	Does the company report the physical risks to its business operations arising from nature loss and ecosystem degeneration?	Nature loss broadly refers to the significant loss of ecosystem functioning, loss of biodiversity or loss of species abundance. Business operations include a company's assets or activities. The risks can extend beyond the company to its value chain. Do not include anything related to climate change mitigation or reducing greenhouse gases. Do not rephrase of the physical nature-related risks mentioned by the company, instead use these in your answer. Answer "Yes" only if the company explicitly defines physical nature-related risks and explains how its business operations would be affected by these physical nature-related risks. Answer "No" if the company only broadly identifies nature risks without clearly explaining how their business operations are affected by these.
11	Does the company systematically report the impact of its business activities on nature?	Do not include anything related to carbon dioxide or methane emissions. Business activities include any operating, financing or investing activities. Answer "No" if the company only broadly outlines its impact for some business activities. Answer "Yes" if the company goes through all of its main business activities explaining how they impact nature. Answer "Yes" if the company explains how it has identified select business activities and explains how they impact nature.
12	Does the company report the dependencies of its business activities on ecosystem services?	Do not include anything related to carbon dioxide or methane emissions. Dependencies outlined should either refer to provisioning ecosystem services (e.g. water provision) or regulation ecosystem services (e.g. water quality). Business activities include any operating, financing or investing activities. Answer "No" if the company does not explicitly refer to these relationships as dependencies.
13	Does the company provide a description of the interconnections between its material impacts and dependencies on nature?	Do not include anything related to carbon dioxide or methane emissions. Business activities include any operating, financing or investing activities. Explanations should link the impacts the company's business activities have on nature with the dependencies the same or other business activities on nature. Answer "No" if the company does not outline any impacts or dependencies on nature. Answer "Yes" if the company explains the ecosystem services that are impacted by its business activities and whether any of its business activities depend on these ecosystem services.
14	Does the company report the location of its material nature-related impacts?	Nature loss broadly refers to the significant loss of ecosystem functioning, loss of biodiversity or loss of species abundance. Business operations include a company's assets or activities. The impacts can extend beyond the company to its value chain. Do not include anything related to climate change mitigation or reducing greenhouse gases. The locations may be disclosed in a map format or a list format. If you find a list of business operations that impact nature with geographical information for the business operations, then answer "Yes". If the company states that it discloses the locations of business activities impacting nature, then answer "Yes". Answer "No" if the company outlines locations or regions affected by the company without identifying the business operations that drive these impacts.
15	Does the company report the locations of it material dependencies?	Nature loss broadly refers to the significant loss of ecosystem functioning, loss of biodiversity or loss of species abundance. Business operations include a company's assets or activities. Dependencies outlined should either refer to provisioning ecosystem services (e.g. water provision) or regulation ecosystem services (e.g. water quality). The

		dependencies can extend beyond the company's direct operations to its value chain. Do not include anything related to climate change mitigation or reducing greenhouse gases. The locations may be disclosed in a map format or a list format. If you find a list of business operations and their ecosystem service dependencies with geographical information for the business operations, then answer "Yes". If the company states that it discloses the locations of business activities that are dependent on ecosystem services, then answer "Yes". Answer "No" if the company outlines locations or regions of their business operations without listing their dependencies on ecosystem services.
16	Does the company disclose the locations of its business activities and assets in or close to ecologically-sensitive areas?	Ecologically sensitive areas are areas that are important for biodiversity, areas of high ecosystem integrity, areas of rapid decline in ecosystem integrity, areas of high physical water risks, and/or areas important for ecosystem services provision. The locations may be disclosed in a map format or a list format. If you find a list of business operations near ecologically sensitive areas with geographical information for the business operations, then answer "Yes". If the company states that it discloses the locations of business activities near ecologically sensitive areas, then answer "Yes". Answer "No" if the company outlines locations or regions that are ecologically sensitive without identifying its business operations affected by these.
17	Does the company identify the anticipated effects of physical nature-related risks on revenues, expenses, cashflows, asset and liability values or funding sources over the short- medium- and long-term?	Answer "Yes" only if the company provides an explanation of the anticipated effects across two or more timescales (i.e. short-, medium or long-term). Answer "No" if the company only explains the anticipated effects in one timescale, i.e. only short-term effects, only medium-term effects, only long-term effects.

Supplementary Table 3. List of indicators for *Risk*.

ID	*Question*	*Definition*
**Assessing climate risk**		
18	Does the company have a specific process in place to identify physical risks arising from climate change?	Physical climate-related risks are a function of climate-related hazards, such as floods, the exposure of assets and operations to these hazards, and the vulnerability of these operations and assets of being disrupted. Do not include carbon or methane emissions as a climate-related risk. Ignore if the company mentions specific physical climate-related risks and instead focus on whether there is a process. Answer "Yes" if a company says it has a process to identify physical climate-related risks or explains this process.
19	Does the company have a specific process in place to identify business opportunities arising from climate change?	Do not include any information related to carbon emission reductions, decarbonisation or greenhouse gases as a climate-related opportunity. Ignore if the company mentions specific climate-related opportunities and instead focus on whether there is a process. Answer "Yes" if a company says it has a process to identify climate-related opportunities or explains this process.
20	Does the company assessment of physical climate-related risks cover its value chain?	Physical climate-related risks are a function of climate-related hazards, such as floods, the exposure of assets and operations to these hazards, and the vulnerability of these operations and assets of being disrupted. Do not include carbon or methane emissions as a climate-related risk. Ignore if the company mentions specific physical climate-related risks and instead focus on whether the process used to identify physical climate-related risks include only direct operations or also its value chain. Answer "Yes" if a company says it has a process to identify physical climate-related risks and explains that this process covers any business activities of its value chain.
21	Does the company report what assumptions it works with when assessing physical risks arising from climate change?	Provide specific examples of the strategic assumptions that the company reports as basis for its physical climate risk assessments. These could include for instance assumptions about the development of consumer preferences, input prices, sector policies, carbon emission trends, applicable climate hazard types and others. Answer "YES" if you find information about the assumptions underlying the physical climate-related risk assessment. Answer "NO" if you do not find this information.
22	Does the company refer to any third-party scenarios when identifying physical climate-related risks (e.g. IPCC trajectories, NGFS scenarios, etc.)?	Physical climate-related risks are a function of climate-related hazards, such as floods, the exposure of assets and operations to these hazards, and the vulnerability of these operations and assets of being disrupted. Provide specific examples of the scenarios, model ensembles and scenario envelopes used by the company to assess its physical climate-related risks. Scenarios can either be from third-parties or generated in-house. Answer "Yes" if a company outlines the scenarios it uses to assess physical climate-related risks.
23	Does the company report the timescales considered when identifying physical climate-related risks and opportunities?	Physical climate-related risks are a function of climate-related hazards, such as floods, the exposure of assets and operations to these hazards, and the vulnerability of these operations and assets of being disrupted. Timescales during physical climate risk assessments explain how many years into the future the company is looking to identify potential physical climate risks. Answer "No" if the company uses only one

		timescale for its physical climate risk assessments or if the company does not mention any timescales. Answer "Yes" if the company explicitly outlines numeric figures for the timescales it uses when identifying physical climate-related risks.
24	Does the company report whether fat-tail risks or tipping points were considered when identifying physical climate-related risks or opportunities?	Physical climate-related risks are a function of climate-related hazards, such as floods, the exposure of assets and operations to these hazards, and the vulnerability of these operations and assets of being disrupted. Example of tipping points include the melting of the ice caps. Fat tail risks are those risks occurring with a lower probability. Answer "No" if the company mentions tipping points but does not explain how these are embedded into its physical climate risk assessment process.
25	Does the company report the frequency at which it carries out assessments of climate-related risks and opportunities?	Physical climate-related risks are a function of climate-related hazards, such as floods, the exposure of assets and operations to these hazards, and the vulnerability of these operations and assets of being disrupted. Do not consider carbon emission reductions or carbon emission generation as a climate-related opportunity or risk. Answer "Yes" only if the company explicitly states the time intervals at which assessments of climate-related risks and opportunities are carried out.
**Assessing nature risk**		
26	Does the company have a specific process in place to identify physical risks arising from nature loss and ecosystem degeneration?	c Do not include carbon or methane emissions as a nature-related risk. Ignore if the company mentions specific physical nature-related risks and instead focus on whether there is a process. Answer "Yes" only if a company states it has a process to identify nature climate-related risks or explains this process.
27	Does the company report to have a specific process in place to analyse its dependencies on ecosystem services?	Business operations include a company's assets or activities. Dependencies outlined should either refer to provisioning ecosystem services (e.g. water provision) or regulation ecosystem services (e.g. water quality). The dependencies can extend beyond the company's direct operations to its value chain. Do not include carbon emission reductions as a dependency. Ignore if the company mentions specific dependencies on ecosystem services and instead focus on whether there is a process. Answer "Yes" only if a company states it has a process to identify dependencies on ecosystem services or explains this process.
28	Does the company report to have a specific process in place to analyse its contributions to nature loss and ecosystem degeneration?	Nature loss broadly refers to the significant loss of ecosystem functioning, loss of biodiversity or loss of species abundance. Do not include carbon emissions as a contribution to nature loss or ecosystem degeneration. Answer "Yes" only if the company outlines a process to measure its contribution to nature loss, ecosystem degeneration or biodiversity loss. Answer "No" if the company only carries out environmental impact assessments without analysing the impact on ecosystem services.
29	Does the company assessment of physical nature-related risks cover its value chain?	Nature loss broadly refers to the significant loss of ecosystem functioning, loss of biodiversity or loss of species abundance. Do not include carbon or methane emissions as a climate-related risk. Ignore if the company mentions specific physical nature-related risks and instead focus on whether the process used to identify physical nature-related risks include only direct operations or also its value chain. Answer "Yes" if a company says it has a process to identify physical nature-related risks and explains that this process covers any business activities of its value chain.

30	Does the company refer to any third-party scenarios when identifying physical nature-related risks (e.g. IPCC trajectories, NGFS scenarios, etc.)?	Nature loss broadly refers to the significant loss of ecosystem functioning, loss of biodiversity or loss of species abundance. Provide specific examples of the scenarios, model ensembles and scenario envelopes used by the company to assess its physical climate-related risks. Scenarios can either be from third parties (e.g. NGFS, IPCC) or generated in-house. Answer "Yes" if a company outlines the scenarios it uses to assess physical nature-related risks.
31	Does the company report the timescales considered when identifying nature-related dependencies, impacts, risks and opportunities?	Timescales during physical nature-related risk assessments explain how many years into the future the company is looking to identify potential physical nature risks. Answer "No" if the company uses only one timescale for its physical nature risk assessments or if the company does not mention any timescales. Answer "Yes" if the company explicitly outlines multiple numeric figures for the timescales it uses when identifying physical nature-related risks.
32	Does the company report whether ecological thresholds or tipping points were considered when identifying nature-related dependencies, impacts, risks and opportunities?	Physical nature risks arise from nature loss, which broadly refers to the significant loss of ecosystem functioning, loss of biodiversity or loss of species abundance. Ecological threshold and tipping points include for example the loss of pollinators. Answer "No" if the company mentions tipping points but does not explain how these are embedded into its physical nature risk assessment process.
33	Does the company report the frequency at which it carries out assessments of nature-related dependencies and impacts?	Do not include carbon emission reductions as a nature-related impact or dependency. Answer "Yes" only if the company explicitly states the time intervals at which assessments of nature-related dependencies and impacts are carried out. Answer "No" if the time interval is only outlined for nature-related impacts.
34	Does the company report the frequency at which it carries out assessments of nature-related risks and opportunities?	Physical nature risks arise from nature loss, which broadly refers to the significant loss of ecosystem functioning, loss of biodiversity or loss of species abundance. Do not consider carbon emission reductions or carbon emission generation as a nature-related opportunity or risk. Answer "Yes" only if the company explicitly states the time intervals at which assessments of nature-related risks and opportunities are carried out.
**Risk management process**		
35	Are the company's processes for identifying, assessing, prioritising and monitoring physical climate- and nature-related risks integrated into its overall risk management process?	Answer "Yes" only if the company explains that climate-related risks and nature-related risks are considered alongside other risks (e.g. operational risks, currency risks, etc.) in risk review meetings or other risk management processes. Answer "No" if the company only mentions one of either climate-related risks or nature-related risks.
36	Does the company report how it determines the relative significance of climate- and nature-related risks to inform its prioritisation of risk responses?	Companies can prioritise certain risks over others by for example estimating their materiality or dealing with higher probability risks. Answer "Yes" only if the company explains the process it uses to prioritise specific risks.

37	Does the company consider multiple physical climate- or nature-related risks occurring in parallel or concurrently?	The relationships of multiple risks occurring can be described as cascading or compounding risks whereby there is overlap in the spatial or temporal dimensions of risks. Answer "Yes" only if the company explicitly mentions concurrent, compounding or cascading risks and explains how it models the impact of multiple physical climate- or nature-related risks occurring at the same time or in quick succession.
38	Does the company intend to improve or expand its location assessment activities over the short-, medium- and long-tern?	Location assessment activities include assessing climate- or nature-related risks in specific geographies rather than generally. Do not include examples of assessments that have been carried out. Instead, focus on content that explicitly outlines how the company intends to improve location assessment activities. Answer "Yes" only if the company sets out plans to improve these assessments across two timescales (i.e. short-, medium- or long-term).
39	Does the company outline areas where it needs to improve data quality for more accurate climate- and nature-related risk assessments?	Climate- and nature-related risk assessments face data constraints. Provide examples where the company acknowledges these data constraints and sets out strategies or initiatives to improve these. Answer "Yes" if the company outlines one or more areas where data quality can be improved for its climate- and nature-related risk assessments.

Supplementary Table 4. List of indicators for *Implementation*.

ID	*Question*	*Definition*
**Operations**		
40	Does the company require a physical climate-related risk or opportunity assessment as part of key business operations? (e.g. procurement, pricing, etc.)	Do not include carbon emission reductions as a climate-related risk or opportunity. Key business operations include activities such as procurement of goods, materials or equipment, pricing during decision-making, or research and development, among others. Provide examples where the company states that it requires physical climate-related risk assessments to be carried out as part of these processes. If you find one r more operations where this is required, answer "Yes". Answer "No" if the company carries out physical climate-related risk assessment but does not state that these are a requirement for specific business operations.
41	Does the company require a physical nature-related risk or opportunity assessment as part of key business operations? (e.g. procurement, pricing, etc.)	Do not include carbon emission reductions as a nature-related risk or opportunity. Key business operations include activities such as procurement of goods, materials or equipment, pricing during decision-making, or research and development, among others. Physical nature-related risk is a function of a hazard (e.g. invasive species), the exposure a business has to the hazard through its business activities and assets, and the vulnerability of business activities and assets or the ecosystem services to those hazards. Provide examples where the company states that it requires physical nature-related risk assessments to be carried out as part of these processes. If you find one or more operations where this is required, answer "Yes". Answer "No" if the company carries out physical nature-related risk assessment but does not state that these are a requirement for specific business operations. Answer "No" if the company carries out environmental impact assessments but does not link these to physical nature-related risks.
42	Does the company report how adjustments to its policies and conditions address specific climate-related risks or opportunities?	Do not include carbon emissions or carbon emission reductions as a climate-related risks or opportunity. Identify examples where the company explicitly links changes it has made or will make to its policies and conditions with physical climate-related risks opportunities it has identified. Answer "No" if the company identifies no climate-related risks or opportunities. Answer "No" if the company outlines some changes but does not link these to physical climate-related risks or opportunities.
43	Does the company report how adjustments to its policies and conditions address specific nature-related risks or opportunities?	Do not include carbon emissions or carbon emission reductions as a nature-related risk or opportunity. Identify examples where the company explicitly links changes it has made or will make to its policies and conditions with physical nature-related risks opportunities it has identified. Answer "No" if the company identifies no nature-related risks or opportunities. Answer "No" if the company outlines some changes but does not link these to physical nature-related risks or opportunities.
44	Does the company report any policies or conditions used to ensure no significant harm is done to nature or ecosystem services?	Policies or conditions could govern its direct operations but also how it engages with its value chain. Find instances where the company has introduced policies and conditions that reduce the probability or potential impact of negatively affecting nature or ecosystem services. The company may refer to Doing No Significant Harm or DNSH. If you find one or more policies and conditions adjusted to include this, answer "Yes".

45	Does the company report any policies or conditions used to ensure no significant harm is done to societal resilience through its business activities?	Policies or conditions could govern its direct operations but also how it engages with its value chain. Find instances where the company has introduced policies and conditions that reduce the probability or potential impact of negatively affecting society, community and people. The company may refer to Doing No Significant Harm or DNSH. If you find one or more policies and conditions adjusted to include this, answer "Yes".
46	Does the company anticipate any significant investments or asset disposals as a result of climate-related risks and opportunities identified?	Do not include carbon emissions or carbon emission reductions as a climate-related risk or opportunity. Answer "Yes" only if the asset disposals or investments are explicitly linked to a physical climate-related risks. Answer "No" if asset disposals or investments are outlined without explaining how these address specific physical climate-related risks.
47	Does the company anticipate any significant investments or asset disposals as a result of nature-related risks and opportunities identified?	Do not include carbon emissions or carbon emission reductions as a nature-related risk or opportunity. Answer "Yes" only if the asset disposals or investments are explicitly linked to a physical nature-related risk. Answer "No" if asset disposals or investments are outlined without explaining how these address specific physical nature-related risks.
48	Does the company explain how climate- and nature-related risks and opportunities are input into financial planning processes?	Do not include carbon emissions or carbon emission reductions as a climate-related or nature-related risk and opportunity. Do not consider specific amounts invested, instead look for evidence where the company integrates nature- or climate-related risks into financial planning. Do not include executive compensation as a financial planning process.
49	Does the company report on the scenario tools and methodologies used to test the resilience of its financial business strategy on climate-related issues?	Climate-related issues include risks and opportunities. Do not include carbon emissions, carbon emission reductions, decarbonisation, greenhouse gas reductions or carbon footprints as a climate-related issue. Scenarios here could include specific emission pathways or one or more risks materialising. If the company explains the type of scenarios used and how they are constructed or how climate-related issues are included in the methodologies used to assess financial resilience, then answer "yes".
50	Does the company report on the scenario tools and methodologies used to test the resilience of its financial business strategy on nature-related issues?	Nature-related issues include risks and opportunities. Do not include carbon emissions, carbon emission reductions, decarbonisation, greenhouse gas reductions or carbon footprints as a nature-related issue. Scenarios here could include specific emission pathways or one or more risks materialising. If the company explains the type of scenarios used and how they are constructed or how nature-related issues are included in the methodologies used to assess financial resilience, then answer "yes".
**Offering**		
51	Does the company refer to external definitions or taxonomies to classify its products and services as resilience aligned?	Answer "Yes" only if the company explains what definitions or taxonomies for resilience it uses to classify its product and service offering.

52	Does the company demonstrate actions taken to adjust its product and service offering to physical climate change risks?	Do not include carbon emissions, carbon emission reductions, decarbonisation, greenhouse gas reductions or carbon footprints. Physical climate-related risks include acute risks such as droughts, floods, wildfires and other weather extremes, as well as chronic risks such as sea level rise.
53	Does the company demonstrate actions taken to adjust its product and service offering to physical nature risks?	Do not include carbon emissions, carbon emission reductions, decarbonisation, greenhouse gas reductions or carbon footprints as a climate-related risk or opportunity. Physical nature-related risks include risks impacting a company's assets or operations directly (i.e. not through policies) arising from nature loss drivers, such as pollution, invasive species or others that affect relevant ecosystem services such as water provision, pollination or others. Do not include physical risks arising from climate, such as droughts, floods, or other weather extremes.
54	Does the company offer or intend to offer products that enable others to respond to physical climate risks?	Do not include carbon emissions, carbon emission reductions, decarbonisation, greenhouse gas reductions or carbon footprints as a climate-related risk or opportunity. Physical climate-related risks include acute risks such as droughts, floods, wildfires and other weather extremes, as well as chronic risks such as sea level rise.
55	Does the company offer or intend to offer products that help regenerate or restore damaged ecosystems?	Ignore anything related to carbon emissions, carbon emission reductions, greenhouse gases or decarbonisation.

Supplementary Table 5. List of indicators for *Engagement*.

ID	*Question*	*Definition*
**with value chain**		
56	Does the company report any current or anticipated changes to upstream sourcing practices and interactions with downstream partners to address physical climate-related risks and opportunities?	Physical climate-related risks arise from acute hazards such as floods, droughts and other, and chronic hazards, such as sea level rise and others. Do not include carbon emissions, carbon emission reductions, decarbonisation or greenhouse gas reductions as a climate-related risk or opportunity. Changes could include, for example, certification practices, requiring risk management processes, or collaboration with suppliers, customers and other stakeholders. If the company has integrated one or more of these, state "yes".
57	Does the company report any current or anticipated changes to upstream sourcing practices and interactions with downstream partners to address physical nature-related issues? (e.g. adoption of improved tracing, certification practices, collaboration with suppliers, customers and other stakeholders, or extended producer responsibility schemes)	Nature-related issues include nature-related dependencies, impacts, risks and opportunities. Do not include carbon emissions, carbon emission reductions, decarbonisation, greenhouse gas reductions, GHG reductions or carbon footprints as a nature-related risk or opportunity. Physical nature-related risks include risks impacting a company's assets or operations directly (i.e. not through policies) arising from nature loss drivers, such as pollution, invasive species or others that affect relevant ecosystem services such as water provision, pollination or others. Changes could include, for example, adoption of improved tracing, certification practices, collaboration with suppliers, customers and other stakeholders, or extended producer responsibility schemes. If the company has integrated one or more of these, state "yes".
58	Does the company report any current or anticipated changes to its engagement processes to incorporate multi-stakeholder planning processes for physical climate-related risks or opportunities?	Climate-related issues include only physical climate-related risks or opportunities. Do not include carbon emissions, carbon emission reductions, decarbonisation, greenhouse gas reductions or carbon footprints. Physical climate-related risks arise from acute hazards such as floods, droughts and other, and chronic hazards, such as sea level rise and others. Multi-stakeholder processes could include landscape approaches, watershed management and marine and coastal spatial planning, among others. Answer "Yes" if the company has integrated multi-stakeholder planning processes for climate-related issues as part of any of its engagements with its value chain.
59	Does the company report any current or anticipated changes to its engagement processes to incorporate multi-stakeholder planning processes for nature-related issues? (E.g. landscape approaches, watershed management and marine and coastal spatial planning)	Nature-related issues include nature-related dependencies, impacts, risks and opportunities. Do not include carbon emissions, carbon emission reductions, decarbonisation, greenhouse gas reductions or carbon footprints as a nature-related risk or opportunity. Multi-stakeholder processes could include landscape approaches, watershed management and marine and coastal spatial planning, among others. Answer "Yes" if the company has integrated multi-stakeholder planning processes for nature-related issues as part of any of its engagements with its value chain.
**with third parties**		

60	Does the company engage with governments and policymakers to manage physical climate-related risks?	Do not include carbon emissions, carbon emission reductions, decarbonisation, greenhouse gas reductions or carbon footprints as a climate-related risk or opportunity. Physical climate-related risks include acute risks such as droughts, floods, wildfires or other weather extremes, as well as chronic risks such as sea level rise and others.
61	Does the company engage with governments and policymakers to manage physical nature-related risks?	Do not include carbon emissions, carbon emission reductions, decarbonisation, greenhouse gas reductions or carbon footprints, as a physical nature risk. Physical nature-related risks include risks impacting a company's assets or operations directly (i.e. not through policies) arising from nature loss drivers, such as pollution, invasive species or others that affect relevant ecosystem services such as water provision, pollination or others. Do not include physical risks arising from climate, such as droughts, floods, or other weather extremes.
62	Does the company report its process for engaging with Indigenous Peoples, Local Communities and affected stakeholders?	Answer "No" if the company only outlines in general terms the importance of engaging with local communities. Answer "Yes" only if the company explains what type of engagement forms it uses to engage with communities and how regularly these are carried out when communities are affected.

Supplementary Table 6. List of indicators for *Governance.*

ID	*Question*	*Definition*
**Institutional governance mechanisms**		
63	Does the company have a climate change adaptation plan?	Answer "Yes" only if the climate change adaptation plan is a stand-alone document or is integrated in its stand-alone transition plan.
64	Does the company report the number or proportion of board members with competence in climate-related issues?	Physical climate-related issues includes risks and opportunities. Do not include carbon emissions, carbon emission reductions, decarbonisation, greenhouse gas reductions or carbon footprints as a physical climate-related issue.
65	Does the company report the number or proportion of board members with competence in nature-related issues?	Nature-related issues include nature-related dependencies, impacts, risks and opportunities. Do not include carbon emissions, carbon emission reductions, decarbonisation, greenhouse gas reductions or carbon footprints as a nature-related issue.
66	Does the company report the processes used through which the board or board committees are informed about physical climate-related risks or opportunities?	Do not include carbon emissions or carbon emission reductions as a climate-related risk or opportunity.
67	Does the company report the processes used through which the board or board committees are informed about nature-related dependencies, impacts, risks or opportunities?	Do not include carbon emissions and carbon emission reductions.
68	Does the company report the frequency at which the board and board committees are informed about climate and nature-related issues?	Do not include carbon emissions and carbon emission reductions. Answer "Yes" only if the time intervals are specified (e.g. quarterly).
69	Does the company report for which decisions the board considers climate- or nature-related issues?	Do not include carbon emissions and carbon emission reductions.
70	Does the company report to use internal or external audit or assurance resources to monitor progress on the goals and targets set to address climate- and nature-related issues?	Do not include carbon emissions and carbon emission reductions as a climate- or nature-related issue.
71	Has the company assigned the responsibilities of assessing and managing climate- and nature-related issues to a management-level position or committee that reports to the board?	Do not include carbon emissions and carbon emission reductions as a climate- or nature-related issue.
72	Does the company provide a description of how human rights due diligence processes, including but not limited to those covering the rights of Indigenous Peoples and Local Communities, are embedded	Answer "No" if the company only outlines in general terms the importance of engaging with local communities. Answer "Yes" only if the company explains how human due diligence processes are embedded across the organisation.

	in the organisation’s strategy, policies, codes of conduct, governance structures or best practices?	
73	Does the company have a mechanism for individuals and communities to raise complaints when they may be adversely affected by the company?	Answer "No" if the company only points to a whistleblower policy without explicitly mentioning a tool or mechanism, such as a portal, webpage or telephone number.
74	Does the company have a policy of non-reprisal against complainants, including human rights defenders, whistle-blowers, and community spokespersons?	Policy of non-reprisal also include sections of policies that prohibit retaliation against complainants. Answer "No" if the company outlines in general terms their commitment to ensuring safety of complainants without mentioning a policy.
75	Does the company have a process in place to escalate any issues that may cause significant harm to climate, nature and society?	Do not include carbon emissions or carbon emission reductions.
**Links**		
76	Does the company have initiatives in place to educate its workforce on climate-related risks?	Do not include carbon emissions or carbon emission reductions.
77	Does the company have initiatives in place to educate its workforce on nature-related risks?	Do not include carbon emissions or carbon emission reductions.
78	Are the company's performance metrics for physical climate-related risks or opportunities included in its remuneration policies?	Provide specific examples of executive management remuneration linked to progress towards achievement of its climate-related targets. Do not include carbon emissions, carbon emission reductions, decarbonisation, greenhouse gas reductions or carbon footprints. Answer "No" if the company has no relevant climate-related metrics. If you find examples, answer "Yes".
79	Are the company's performance metrics for nature-related dependencies, impacts, risks or opportunities included in its remuneration policies?	Provide specific examples of executive management remuneration linked to progress towards achievement of its nature-related targets. Do not include carbon emissions, carbon emission reductions, decarbonisation, greenhouse gas reductions or carbon footprints. Answer "No" if the company has no relevant nature-related metrics. If you find relevant examples, answer "Yes".

Supplementary Table 7. List of indicators for *Metrics & Targets*.

ID	*Question*	*Definition*
**Targets**		
80	Does the company set short-, mid- and long-term targets for reducing its exposure to physical climate risks?	Do not include targets related to carbon emissions, carbon emission reductions, decarbonisation, greenhouse gas reductions or carbon footprints. Answer "Yes" if the company provides targets in two or more timescales (e.g. short-term and long-term, or short-term and medium-term).
81	Does the company set short-, mid- and long-targets to improve or maintain ecosystem services?	Do not include targets related to carbon emissions, carbon emission reductions, decarbonisation, greenhouse gas reductions or carbon footprints. Answer "Yes" if the company provides targets in two or more timescales (e.g. short-term and long-term, or short-term and medium-term).
82	Does the company set targets to halt and reverse nature loss and improve or maintain the state of nature?	Nature loss broadly refers to the significant loss of ecosystem functioning, loss of biodiversity or loss of species abundance. Do not include targets related to carbon emissions, carbon emission reductions, decarbonisation, greenhouse gas reductions or carbon footprints.. Answer "yes" only if the company sets its own targets. Answer "No" if the company only mentions targets with explicitly defining these.
83	Does the company provide an explanation of how its climate adaptation-related targets align with the Sharm-el-Sheikh Adaptation Agenda or other similar adaptation goals?	
84	Does the company provide an explanation of how its nature-related targets align with the Kunming-Montreal Global Biodiversity Framework or other similar environmental treaties?	Do not include targets related to carbon emissions, carbon emission reductions, decarbonisation, greenhouse gas reductions or carbon footprints. Answer "Yes" only if the company defines what the third-party goals and targets are. Answer "No" if the company has no relevant nature-related targets.
85	Does the company establish baselines for its climate- and nature-related targets?	Do not include targets related to carbon emissions, carbon emission reductions, decarbonisation, greenhouse gas reductions or carbon footprints. Baselines can be set either in the past or at current standards. Identify all climate- and nature-targets and count the targets with baselines. Answer "Yes" if over half of the targets have baselines. Answer "No" if the company sets not relevant climate- or nature-related targets.
86	Are the company's climate- and nature-related targets validated by an independent third party?	Do not include targets related to carbon emissions, carbon emission reductions, decarbonisation, greenhouse gas reductions or carbon footprints. Answer "No" if the company sets no relevant climate- or nature-related targets.
**Metrics**		
87	Does the company quantify its climate- and nature-related targets using metrics?	Do not include targets related to carbon emissions, carbon emission reductions, decarbonisation, greenhouse gas reductions or carbon footprints. Answer "No" if the company sets no relevant climate- or nature-related targets.

88	Does the company report any metrics for changes to state of nature or changes in the availability of ecosystem services for any of its listed locations?	Do not include metrics related to carbon emissions, carbon emission reductions, decarbonisation, greenhouse gas reductions or carbon footprints. Listed locations are the locations the company has identified are important to its operations either due to ecological sensitivity of the site, the impact or dependency of its business activities on nature at that location, or any other reason the site may be important from the perspective of nature-related issues.
89	Does the company have metrics in place to monitor the financial effects of climate- and nature-related issues on the organisation?	Do not include metrics related to carbon emissions, carbon emission reductions, decarbonisation, greenhouse gas reductions or carbon footprints.
90	Does the company report its climate adaptation-aligned capital expenditure?	Do not include capital expenditure related to carbon emissions, carbon emission reductions, decarbonisation, greenhouse gas reductions or carbon footprints. Answer "Yes" only if the company explains how it defines climate adaptation alignment for its capital expenditure and then lists its values.
91	Does the company report its nature-aligned capital expenditure?	Answer "Yes" only if the company explains how it defines nature alignment for its capital expenditure and then lists its values.

Supplementary Table 8

Notes: The percentages represent the proportion of indicators reported on by all companies in our sample, disaggregated by disclosure elements.

		Disclosure elements					
	All indicators	Foundation	Risk	Implementation	Engagement	Governance	Metrics & Targets
Mean	20.4%	11.0%	18.3%	29.3%	25.1%	31.5%	7.1%
Min.	1.1%	0%	0%	0%	0%	0%	0%
Max.	54.9%	64.7%	72.7%	81.3%	85.7%	64.7%	41.7%
St. dev.	9.8%	11.1%	14.2%	16.6%	18.8%	13.4%	10.1%

Supplementary Note 1

Notes: This table shows the prompt template for the LLM, where “basic_info” is the basic information about a company, i.e. name, sector, and location, “sources” is the top-10 retrieved information that is used to answer the question, “question” is the question to answer, “explanation” is an optional additional explanation of the question, and “answer_length” is the length restriction for the answer.

```
"""
You are a senior sustainability analyst with expertise in climate science evaluating a company's climate-related transition plan and strategy.

This is basic information to the company:
{basic_info}

You are presented with the following sources from the company's report:
--------------------- [BEGIN OF SOURCES]\n
{sources}\n
--------------------- [END OF SOURCES]\n

Given the sources information and no prior knowledge, your main task is to respond to the posed question encapsulated in "||".
Question: ||{question}||

Please consider the following additional explanation to the question encapsulated in "+++++" as crucial for answering the question:
+++++ [BEGIN OF EXPLANATION]
{explanation}
+++++ [END OF EXPLANATION]

Please enforce to the following guidelines in your answer:
1. Your response must be precise, thorough, and grounded on specific extracts from the report to verify its authenticity.
```

2. If you are unsure, simply acknowledge the lack of knowledge, rather than fabricating an answer.
3. Keep your ANSWER within {answer_length} words.
4. Be sceptical to the information disclosed in the report as there might be greenwashing (exaggerating the firm's environmental responsibility). Always answer in a critical tone.
5. Cheap talks are statements that are costless to make and may not necessarily reflect the true intentions or future actions of the company. Be critical for all cheap talks you discovered in the report.
6. Always acknowledge that the information provided is representing the company's view based on its report.
7. Scrutinize whether the report is grounded in quantifiable, concrete data or vague, unverifiable statements, and communicate your findings.
8. Start your answer with a "[[YES]]"" or ""[[NO]]"" depending on whether you would answer the question with a yes or no. Always complement your judgement on yes or no with a short explanation that summarizes the sources in an informative way, i.e. provide details.

Format your answer in JSON format with the two keys: ANSWER (this should contain your answer string without sources), and SOURCES (this should be a list of the SOURCE numbers that were referenced in your answer).
Your FINAL_ANSWER in JSON (ensure there's no format error):
"""
